# Tracheal agenesis diagnosed by supraglottic airway–assisted endoscopic evaluation during neonatal resuscitation: a case report

**DOI:** 10.1186/s40981-026-00852-w

**Published:** 2026-02-05

**Authors:** Erika Miyazaki, Kentaro Yamakawa, Marie Kusumoto, Yoshie Taniguchi, Shoichi Uezono

**Affiliations:** The Department of Anesthesiology, The Jikei University School of Medicine, 3-25-8 Nishi-shimbashi, Minato-ku, Tokyo, 105-8461 Japan

**Keywords:** Tracheal agenesis, Supraglottic airway, Fiberoptic bronchoscopy, Tracheoesophageal fistula, Neonatal airway management

## Abstract

**Background:**

Tracheal agenesis is an exceptionally rare and typically lethal congenital airway anomaly presenting with failed intubation. We report a case of unrecognized tracheal agenesis in which supraglottic airway-assisted ventilation enabled concurrent fiberoptic endoscopic evaluation during neonatal resuscitation.

**Case presentation:**

A preterm male neonate born at 31 weeks and 1 day of gestation with polyhydramnios and suspected duodenal atresia developed severe respiratory failure immediately after delivery. Repeated attempts at tracheal intubation were unsuccessful despite vocal cord–like structures being seen. Placement of a size 0.5 supraglottic airway (Air-Q^®^) resulted in transient partial ventilation with intermittent colorimetric carbon dioxide detection, permitting fiberoptic endoscopic evaluation. Endoscopy demonstrated a blind-ending subglottic pouch and a distal tracheoesophageal fistula, strongly suggesting tracheal agenesis; computed tomography subsequently confirmed the diagnosis of Floyd type II tracheal agenesis.

**Conclusions:**

Early supraglottic airway-assisted endoscopic evaluation may facilitate rapid recognition of tracheal agenesis during neonatal resuscitation and support timely, multidisciplinary decision-making after failed intubation.

**Supplementary Information:**

The online version contains supplementary material available at 10.1186/s40981-026-00852-w.

## Background

Tracheal agenesis is an exceptionally rare and typically lethal congenital airway anomaly, with an estimated incidence of approximately 1 in 100,000 births [1]. Tracheal agenesis usually presents at delivery with severe respiratory failure, characterized by failed tracheal intubation and ineffective or unsustainable ventilation due to tracheal absence or discontinuity. In most cases, a tracheoesophageal fistula allows limited esophageal ventilation, which is insufficient to sustain oxygenation [2]. This partial airway patency may obscure the characteristic prenatal features of congenital high airway obstruction syndrome, making antenatal diagnosis uncommon and challenging [1, 3].

Current neonatal resuscitation guidelines recommend early use of a supraglottic airway when intubation is unsuccessful [4–6]. In the presence of a tracheoesophageal fistula, detection of exhaled carbon dioxide during resuscitation does not necessarily indicate effective pulmonary ventilation. It may create a misleading impression of airway patency, particularly when supraglottic airways are used as rescue devices after failed intubation.

Supraglottic airways are commonly used for elective airway management; they also serve as rescue devices in neonatal resuscitation algorithms. They can provide a conduit for diagnostic fiberoptic evaluation when direct laryngoscopy fails [7, 8]. However, their diagnostic application in congenital upper airway obstruction, particularly in preterm neonates, has rarely been described. We report a preterm neonate in whom early supraglottic airway-assisted endoscopic assessment allowed for rapid anatomical recognition of tracheal agenesis and guided urgent decision-making during neonatal resuscitation.

## Case presentation

A male neonate was delivered at 31 weeks and 1 day of gestation by emergency cesarean section for threatened preterm labor. Prenatal ultrasound demonstrated polyhydramnios and a double-bubble sign suggestive of duodenal atresia, with no airway abnormality detected. Birth weight was 2,213 g. At delivery, the neonate was apneic, cyanotic, and bradycardic (approximately 70 beats per minute), with Apgar scores of 2 at 1 min and of 4 at 5 min, respectively.

Pulse oximetry became available approximately 3 min after birth, revealing an oxygen saturation of 45% (Table 1). Tracheal intubation was attempted repeatedly; vocal cord–like structures could be seen, no identifiable glottic opening was present, and the tracheal tube could not be advanced beyond a firm, membrane-like obstruction. Oxygen saturation remained low during three failed tracheal intubation attempts (43% at 4.5 min, with a heart rate of approximately 70 beats per minute).

**Table 1:** Timeline of clinical events

Time from birth	Clinical event	Key findings/interpretation
0 min	Birth	Gestational age 31 + 1 weeks; apnea immediately after birth
~ 3 min	Pulse oximetry available	SpO₂ 45%
~ 4.5 min	Tracheal intubation attempt (three attempts)	No effective ventilation was achieved
~ 6 min	Bag-mask ventilation	Transient increase in SpO₂, not sustained
~ 10 min	Arrival of the pediatric anesthesia team	A supraglottic airway device was placed
Immediately after	Fiberoptic airway evaluation	No tracheal lumen was seen, and the airway anatomy was inconsistent with tracheal patency
Within 2 h	Computed tomography	Floyd type II tracheal agenesis
~ 6 h	Outcome	Death despite ongoing resuscitative efforts

Intermittent CO₂ detection during resuscitation was observed with a colorimetric detector and was interpreted as limited and transient gas exchange rather than evidence of effective pulmonary ventilation

*SpO*₂ Peripheral oxygen saturation

Bag-mask ventilation at 6 min resulted in a transient increase in heart rate to approximately 150 beats per minute and oxygen saturation to 86%. Given the fixed subglottic obstruction encountered during intubation, congenital upper airway obstruction was suspected, and the pediatric anesthesia team was urgently consulted.

**Fig. 1:**
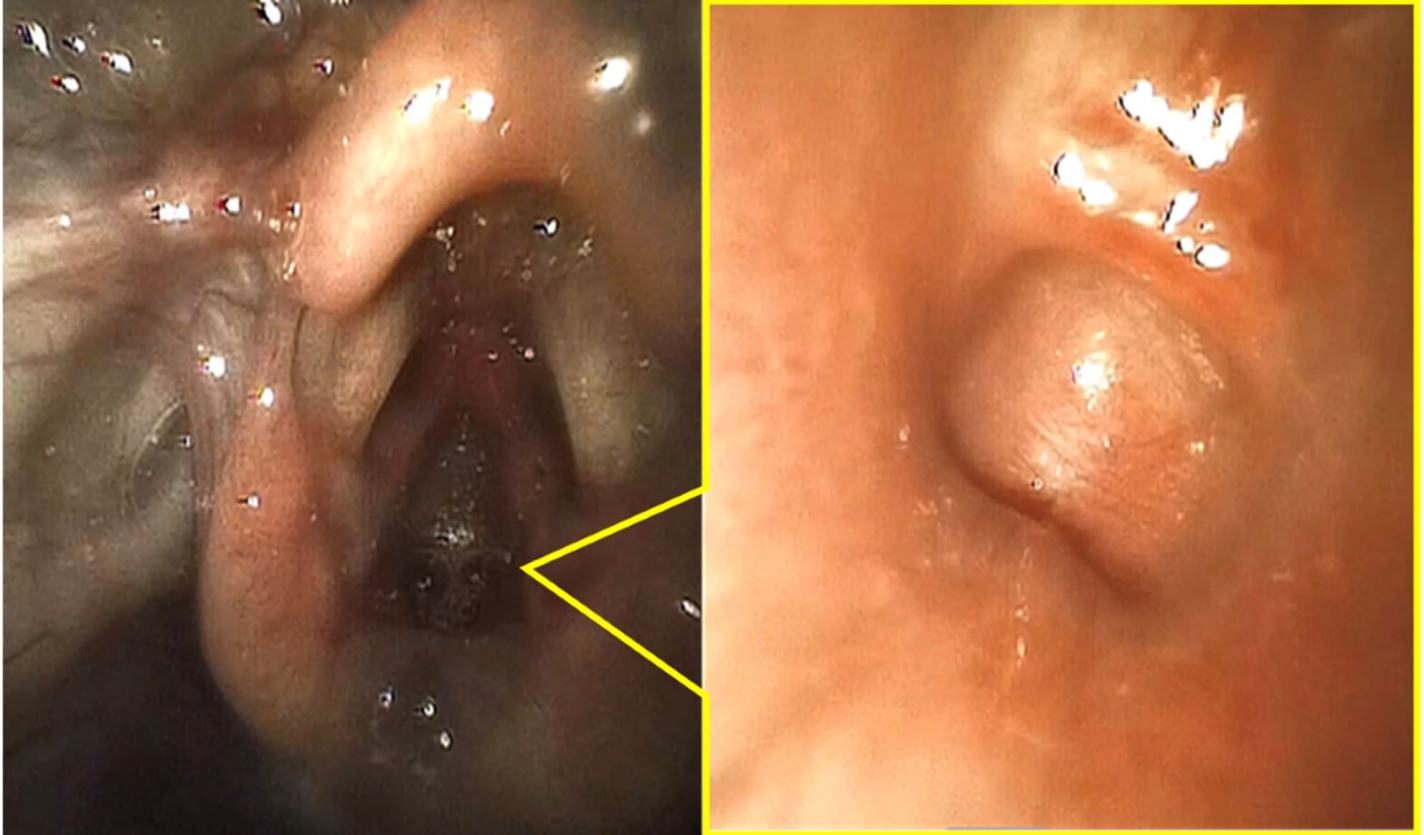
༎Bronchoscopic findings through the supraglottic airway (Left: glottis-like structure; Right: blind-ending subglottic pouch)

**Fig. 2:**
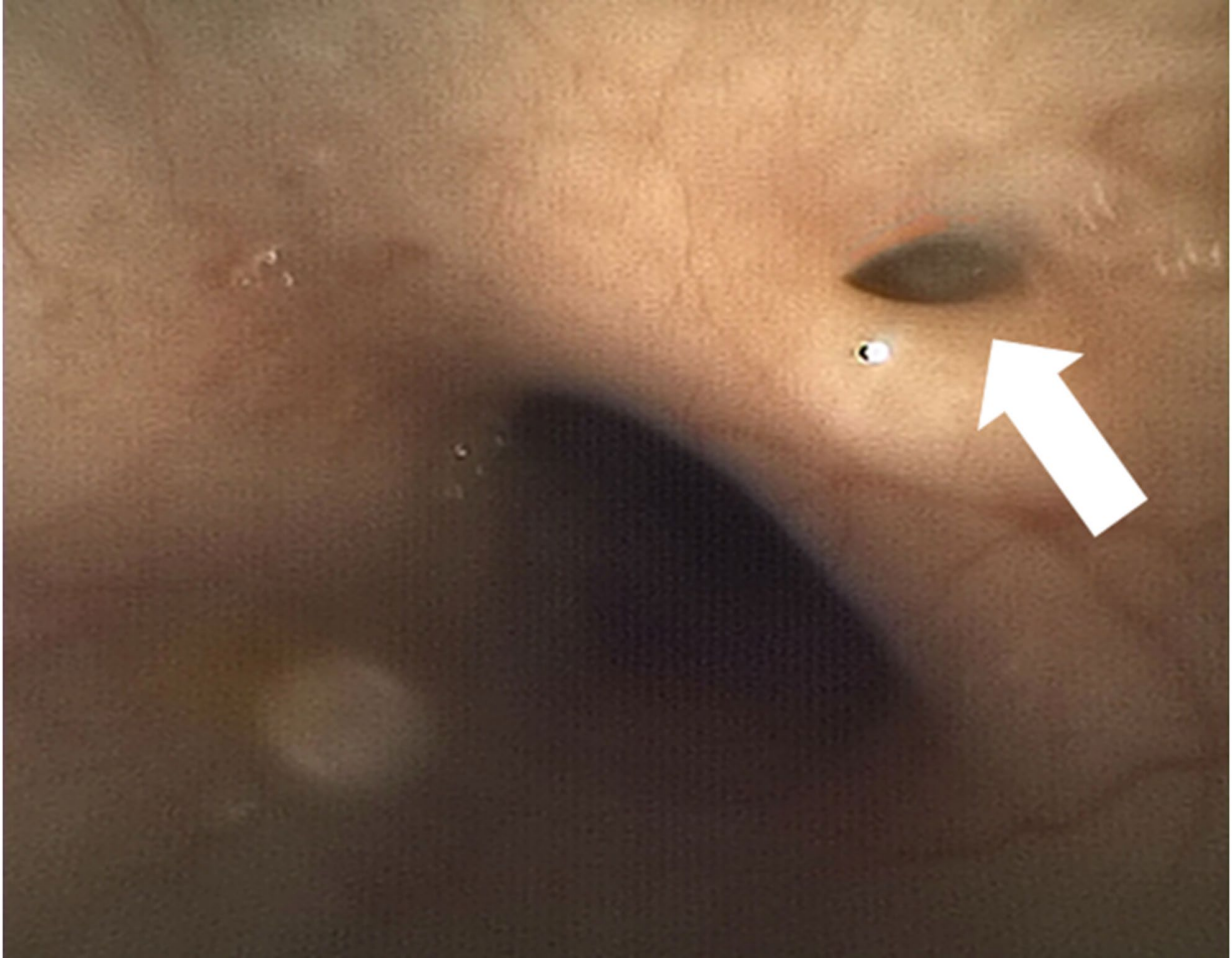
༎Dilated esophagus and tracheoesophageal fistula (arrow)

**Fig. 3:**
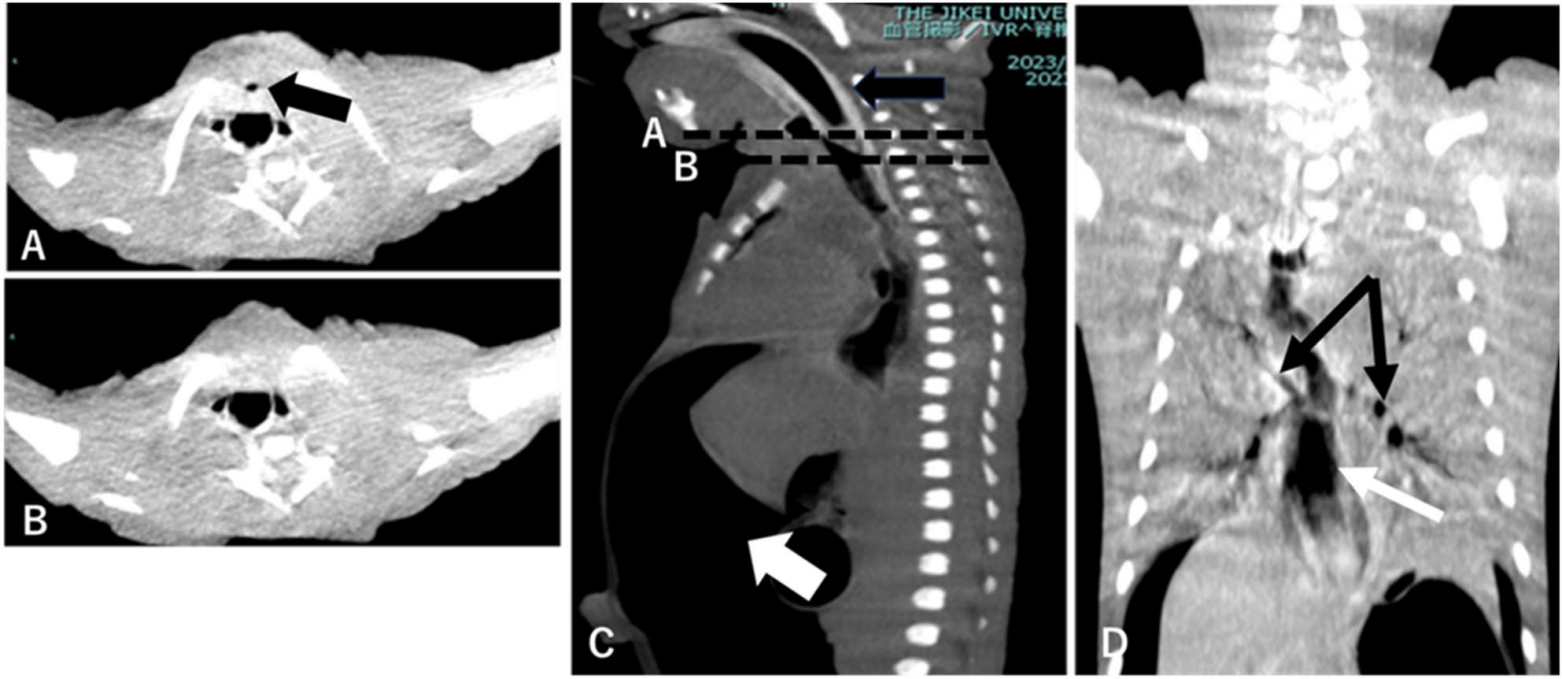
༎ Neck and chest computed tomography. **A **Axial neck computed tomography at the level indicated by line **A **on the sagittal view **C**. The black arrow indicates the blind-ending upper airway subglottic pouch. **B** Axial neck computed tomography at the level indicated by line **B **on the sagittal view (**C**), demonstrating absence of a patent tracheal lumen. **C** Sagittal cervicothoracic computed tomography. The black arrow shows the supraglottic airway in situ; the white arrow indicates a large volume of free intraperitoneal air. **D **Coronal cervicothoracic computed tomography demonstrating bilateral main bronchi (black arrows) and markedly dilated esophagus (white arrow), consistent with gas insufflation

Upon arrival of the pediatric anesthesia team (approximately 10 min after birth), a size 0.5 supraglottic airway (Air-Q^®^) was placed on the first attempt, resulting in limited and transient gas exchange, evidenced by intermittent colorimetric carbon dioxide detection, which was insufficient to sustain effective pulmonary ventilation. With ventilation temporarily stabilized, fiberoptic bronchoscopy with an outer diameter of 2.2 mm was performed immediately thereafter through the supraglottic airway. A blind-ending pouch could be seen immediately below the vocal cord–like structure (Fig. 1). Advancement of the bronchoscope via the esophageal inlet demonstrated a small fistulous opening consistent with a tracheoesophageal fistula (Fig. 2), raising strong suspicion for tracheal agenesis (Fig. 3).

Because of intermittent ventilatory instability and the possibility of distal airway patency, emergent tracheostomy was considered; point-of-care ultrasonography could not reliably identify a patent tracheal lumen. Surgical exploration failed to identify a patent trachea, and tracheostomy could not be performed. Computed tomography was performed within the first two hours of life and confirmed Floyd type II tracheal agenesis. Severe gastric distension was observed early after the initiation of positive-pressure ventilation. In the absence of a patent trachea, insufflated gas predominantly entered the esophagus, with only limited ventilation of the distal airway through the fistula. In the setting of prenatally suspected duodenal atresia, progressive gastric overdistension resulted in gastric perforation and pneumoperitoneum. Given the absence of feasible airway reconstruction and the uniformly fatal prognosis, further life-prolonging interventions were deemed nonbeneficial. Palliative care was initiated, and the neonate died approximately six hours after birth.

## Discussion

In neonates with tracheal agenesis, partial ventilation mediated through a tracheoesophageal fistula may produce transient chest movement and detectable carbon dioxide, creating a misleading impression of airway patency. Because tracheal agenesis is exceedingly rare, such findings during acute neonatal resuscitation may delay recognition of complete airway absence and obscure the underlying diagnosis.

When tracheal intubation is unsuccessful despite appropriate technique, repeated attempts in the presence of a fixed obstruction below the vocal cords should raise suspicion for congenital structural airway abnormalities rather than technical difficulty alone. In such situations, other upper airway obstructive disorders may be preferentially considered, further complicating timely diagnosis.

From an anatomical and physiological perspective, limited ventilation through a tracheoesophageal fistula in tracheal agenesis can be explained by known physiological mechanisms. Previous reports have described cases in which accidental or unintentional esophageal intubation resulted in transient improvement in oxygenation, retrospectively prompting suspicion of tracheal agenesis with tracheoesophageal fistula [1, 2, 9]. However, intentionally selecting esophageal ventilation as a management strategy during neonatal resuscitation is clinically challenging, as esophageal intubation is generally regarded as an adverse event and is difficult to justify for a rare condition.

In this setting, supraglottic airway placement may permit partial airflow into the esophagus, which, in the presence of a tracheoesophageal fistula, can result in limited ventilation evidenced by transient chest movement and detectable carbon dioxide. Although isolated reports have described the use of supraglottic airways, including laryngeal mask airways, in neonates with tracheal agenesis, these devices have primarily been used for temporary oxygenation or stabilization rather than for diagnostic purposes [7, 8, 10]. In contrast, in the present case, partial ventilation achieved after supraglottic airway placement enabled fiberoptic endoscopic assessment, revealing a blind-ending subglottic cavity and a distal tracheoesophageal fistula. Under time-critical resuscitation conditions, supraglottic airway placement functioned as a practical diagnostic bridge rather than a definitive ventilatory strategy.

Ventilation through a tracheoesophageal fistula is also associated with gastrointestinal complications, particularly when gastric decompression is limited. In the present case, concomitant duodenal atresia and evidence of gastrointestinal perforation indicated that continued positive-pressure ventilation carried a high risk of further deterioration. This course underscores the limitation of assessing reversibility or prognosis based solely on partial ventilation in tracheal agenesis complicated by gastrointestinal obstruction.

Although theoretical strategies combining limited ventilation and gastric decompression have been described in highly selected cases, such approaches are not established and depend on favorable anatomical conditions. Accordingly, given the absence of reconstructive options and concern for gastrointestinal perforation, care was redirected to palliative management following multidisciplinary discussion with the family. Earlier reports have described the use of gastrostomy connected to an underwater seal system in selected infants with esophageal atresia and distal tracheoesophageal fistula [11]; this approach has been reported only in isolated cases and is applicable only in exceptional circumstances. These limitations underscore the importance of early anatomical diagnosis to support timely, individualized decision-making in this otherwise uniformly fatal condition.

## Supplementary Information

Supplementary Material 1.

## Data Availability

Not applicable.
