# Supplementary material for: Tracheal agenesis diagnosed by supraglottic airway–assisted endoscopic evaluation during neonatal resuscitation: a case report
Source: JA Clin Rep. 2026 Feb 5;12:16. doi: 10.1186/s40981-026-00852-w (PMC12972372; doi:10.1186/s40981-026-00852-w)
Supplement: Supplementary file 1 — Supplementary Material 1. [file 40981_2026_852_MOESM1_ESM.docx]

December 25, 2025

Shinju Obara, MD, PhD.

Editor-in-Chief

JA Clinical Report

Re: Manuscript title:

**Tracheal agenesis diagnosed by supraglottic airway–assisted endoscopic evaluation during neonatal resuscitation: a case report.**

Dear Dr. Obara,

Tracheal agenesis (TA) is an exceptionally rare and typically fatal congenital airway anomaly that often presents with failed endotracheal intubation at birth. In this report, we describe a preterm neonate in whom placement of a supraglottic airway (SGA) after failed intubation allowed transient partial ventilation and facilitated emergent bronchoscopic evaluation. This led to rapid identification of a blind-ending subglottic cavity and a distal tracheoesophageal fistula, enabling timely diagnosis of Floyd type II TA and informed multidisciplinary decision-making.

Importantly, the SGA was not used as a definitive therapeutic airway but functioned as a diagnostic bridge during neonatal resuscitation. We believe the diagnostic role of SGA-assisted endoscopic evaluation in unanticipated congenital airway obstruction is rarely reported, particularly in preterm neonates, and may provide clinically relevant insights for readers of JA Clinical Reports.

This manuscript has not been published previously and is not under consideration elsewhere. All authors have approved the final manuscript and comply with the journal’s ethical and editorial policies.

Sincerely yours,

Kentaro Yamakawa, MD

The department of Anesthesiology,

The Jikei University School of Medicine

Tokyo, Japan

Email: KYamakawa@jikei.ac.jp
